# einstein (São Paulo) an innovative way to publish your research

**DOI:** 10.1590/S1679-45082018ED4487

**Published:** 2018-04-06

**Authors:** Edna Terezinha Rother

**Affiliations:** 1 Hospital Israelita Albert Einstein, São Paulo, SP, Brazil.

Publishing in scientific journals is still the format most used by researchers to communicate their research findings. Changes in how scientific information is produced and consumed, along with innovations in science communication and diffusion, have influenced the need to promote a paradigm shift in the format and timing of publications, and such changes directly affect scientific journals. Today, journals cannot afford long periods to publish authors’ contributions or to ignore the needs of those who produce knowledge, nor can they be unaware of technology advances and movements in communication networks that allow interoperability among different systems and repositories in which publications are stored and indexed.

Despite the great advances of the last decade, such as electronic-format publication, the open access movement, and “ahead of print” publication, publishing an article is still a demanding time for journals that publish articles within a pre-established period based on frequency or periodicity.

Recently, many high-prestige international journals, *e.g.*, PLoSOne, British Medical Journal, and the Journal of Cell Science, have moved to a continuous article publishing (CAP) workflow. This format allows immediate publication of articles after the review and approval process. This continuous workflow is an innovative way of publishing because in addition to making research results available faster, it enables faster communication between research and researchers and provides interoperability advantages between different systems and communication networks.

## THE NEW MODEL OF PUBLISHING OF EINSTEIN (SÃO PAULO)

Starting in 2018, **einstein** (São Paulo) is moving to a new model of publishing, continuous publication. However, we will continue to produce, although at a reduced frequency, our print version. Our “ahead of print” model will no longer exist.

This new model will bring a number of changes. The journal will have a new and user-friendly website to improve navigation and interoperability; in addition, continuous publication will enable immediate sharing of articles in social and scientific networks.

A publication date will become mandatory for all articles. Therefore, all readers will be able to navigate throughout the journals’ content based on an article’s publication date (*i.e.*, from newer to older articles). Previous issues of our journal will also made available in the new platform.

Another important aspect in this changing is how articles will be cited. All articles will continue to receive a Digital Object Identifier (DOI). However, pages will be represented by e-location, which is an exclusive identification of article pages in the citation. Pages in the bibliographic citation will no longer be based on print version.

Each citation will have year, volume, issue and e-location. This format will appear in the PDF version of each article of our journal in MEDLINE, PubMed, Scopus, Scielo, Emerging Citation Index (Web of Science) and all other bibliographic databases in which our journal is indexed.

Authors publishing in our journal will receive an alert notifying them about publication of their contribution.

After some years of editorial experience, and solid understanding of editorial responsibilities and the means and mechanisms that go into producing a scientific publication, I am sure that this structural changes in **einstein** (São Paulo) will not only positively impact the competitiveness of our journal, but it will also increase the journal’s potential to better and more quickly address the needs of our readers and authors, and the science they engage in.

